# Dynamic changes in DNA methylation during seahorse (*Hippocampus reidi*) postnatal development and settlement

**DOI:** 10.1186/s12983-021-00436-7

**Published:** 2021-10-09

**Authors:** Paula Suarez-Bregua, Sofia Rosendo, Pilar Comesaña, Lucia Sánchez-Ruiloba, Paloma Morán, Miquel Planas, Josep Rotllant

**Affiliations:** 1 Acuatic Biotechnology-ACUABIOTEC Lab, Department of Biotechnology and Aquaculture, Marine Research Institute IIM-CSIC, Vigo, Pontevedra, Spain; 2 Department of Biochemistry, Genetics and Immunology, University of Vigo, Pontevedra, Spain; 3 Department of Ecology and Marine Resources, Marine Research Institute IIM-CSIC, Vigo, Pontevedra, Spain

**Keywords:** Seahorse, *Hippocampus*, DNA methylation, Settlement, MSAP analysis

## Abstract

**Introduction:**

Most living marine organisms have a biphasic life cycle dependent on metamorphosis and settlement. These critical life-history events mean that a developmentally competent larva undergoes a range of coordinated morphological and physiological changes that are in synchrony with the ecological transition from a pelagic to a benthonic lifestyle. Therefore, transition from a pelagic to a benthonic habitat requires multiple adaptations, however, the underlying mechanisms regulating this process still remains unclear. Epigenetic regulation and specifically DNA methylation, has been suggested to be particularly important for organisms to adapt to new environments. Seahorses (Family *Syngnathidae*, Genus *Hippocampus*) are a fascinating group of fish, distinguished by their unique anatomical features, reproductive strategy and behavior. They are unique among vertebrate species due to their “male pregnancy”, where males nourish developing embryos and larvae in a brood pouch until hatching and parturition occurs. After birth, free-swimming offspring are pelagic and subsequently they change into a demersal lifestyle. Therefore, to begin to address the question whether epigenetic processes could be involved in the transition from a planktonic to a benthonic lifestyle observed in seahorses, we studied global DNA methylation profiles in a tropical seahorse species (*Hippocampus reidi*) during postnatal development and settlement.

**Results:**

We performed methylation-sensitive amplified polymorphism (MSAP) along with quantitative expression analysis for genes suggested to be involved in the methylation machinery at six age groups: 1, 5, 10, 20, 30 and 40 days after male’s pouch release (DAR). Results revealed that the *H. reidi* genome has a significantly different DNA methylation profile during postnatal development and settlement on demersal habitats. Moreover, gene expression analysis showed up- and down-regulation of specific DNA methyltransferases (DNMTs) encoding genes.

**Conclusion:**

Our data show that the differences in the DNA methylation patterns seen among developmental stages and during the transition from a pelagic to a benthonic lifestyle suggest a potential for epigenetic regulation of gene expression (through DNA methylation) in this species. Therefore, epigenetic mechanisms could be necessary for seahorse settlement. Nevertheless, if these epigenetic mechanisms come from internal or if they are initiated via external environmental cues should be further investigated.

## Background

Seahorses are marine teleost fish belonging to the genus *Hippocampus* (Family *Syngnathidae)*. Syngnathids are characterized by having biological and ecological traits unique in the animal kingdom including their body structure and the reproductive mode termed “male pregnancy” [1, 2]. Males, rather than females, gestate developing embryos and larvae in a specialized brood pouch [3]. Hatching occurs inside the paternal brood pouch and larvae are released to the aquatic environment after parturition [4, 5]. As other teleost species, seahorses have a biphasic life cycle [6]. Newborn offspring are free-swimming larvae that shift from pelagic (*i.e.,* water column) to demersal or benthonic (*i.e.,* near or on the bottom) habitats [7, 8]. Upon release, seahorses experience some morphological and physiologic changes which become them in individuals ready for settlement and adaptation to a new lifestyle on the bottom of the sea [5, 9]. To date most developmental studies have examined in vivo the early life-history stages of seahorses [5, 10–12] and used histological approaches to describe the tissues and organs formation across ontogeny [13–15]. Recently, de novo genome assembly in *Hippocampus comes* and comparative genomic analysis along with gene expression [16] and transcriptomic studies [17] also revealed some molecular factors involved in the unusual morphological and reproductive features of seahorses. However, little attention has focused on molecular mechanisms governing the postnatal development linked to life style change that all *Hippocampus* species experience [18].

DNA methylation is one of the most studied epigenetic mechanisms that plays an essential role in vertebrate development and ontogeny [19, 20] as the establishment of specific genome methylation patterns contributes to gene expression regulation. Genome methylation mostly consists in the methyl group addition to C5 position of the cytosine ring of DNA (*i.e.,* 5 methylcytosine—5mC) [21]. Methyl groups are added to cytosine residues by the DNA methyltransferases (DNMTs). DNMTs are the writers of the methylome, as DNMT1 is involved in maintaining existing methylation status and DNMT3A/DNMT3B are responsible for de novo methylation [21–23].Simply put, DNA methylation leads to the repression of gene expression while demethylation is linked to transcriptional activation. In fish, changes in genome methylation pattern were shown to contribute to important developmental and physiological processes related to the ecological transition to a new habitat. Epigenetics was shown to play a key role in trout smoltification [24, 25]. Smoltification is a seawater adaptation process to complete the downstream migration from rivers to the ocean that salmonids experience across their life cycle. Genome-wide methylation changes were observed in gills of brown trout (*Salmo trutta*) fed an enriched-salt diet, which triggered the branchial tissue adaptation to seawater environment [24]. Also, variation in the DNA methylation pattern was found between the seawater and freshwater rainbow trout (*Oncorhynchus mykiss*) morphotypes [25]. Metamorphosis is another outstanding developmental event where a competent larva is remodeled into a juvenile and then recruited to its adult habitat [26]. This process usually implies the acquisition of new morphological, physiological and behavioral features for the adaptation to a new stage of the life cycle in a different environment [27]. In this context, global DNA methylation-mediated epigenetic changes were associated with metamorphosis and environmental transitions in European eel (*Anguilla anguilla*) [28] and lamprey (*Petromyzon marinus*) [29]. In Japanese flounder (*Paralichthys olivaceus*), the DNA methylation pattern of muscle-related genes (*smyd1a* and *smyd1b*) was associated to the modulation of their gene expression levels during metamorphosis [30]. However, genome methylation analysis in turbot (*Scophthalmus maximus*) post-embryonic development showed that epigenetic changes seem to correlate with chronological ages rather than specific developmental stages [31].

Seahorses are considered highly vulnerable species to the anthropogenic threats including overfishing for commercial purposes (*i.e.,* aquarium trade and traditional Chinese medicine), bycatch and habitat destruction [32, 33]. This is largely due to their biological traits such as low mobility, low fecundity, long parental care and mate fidelity in most species, among others. In the last decades the real decline of wild populations has led *hippocampus* species to be listed on IUCN Red List of Threatened Species [34] and Appendix II of CITES (Convention on International Trade in Endangered Species of Wild Fauna and Flora) [35]. Recently, syngnathid aquaculture has emerged as a good alternative to stop the massive captures of these animals and promote the conservation of the endangered natural populations [36–38]. Therefore, understanding seahorse biology and behavior as well as the underlying molecular basis becomes particularly important to achieve a successful and economically profitable aquaculture. In the present study, we wonder whether epigenetic mechanisms could be associated to seahorse postnatal development and ecological transition to the demersal habitat. Thus, we characterized the global DNA methylation pattern in *H. reidi* across six age groups after birth using methylation-sensitive amplified polymorphism (MSAP) analysis.

## Results

### Seahorse development and growth

**Fig. 1:**
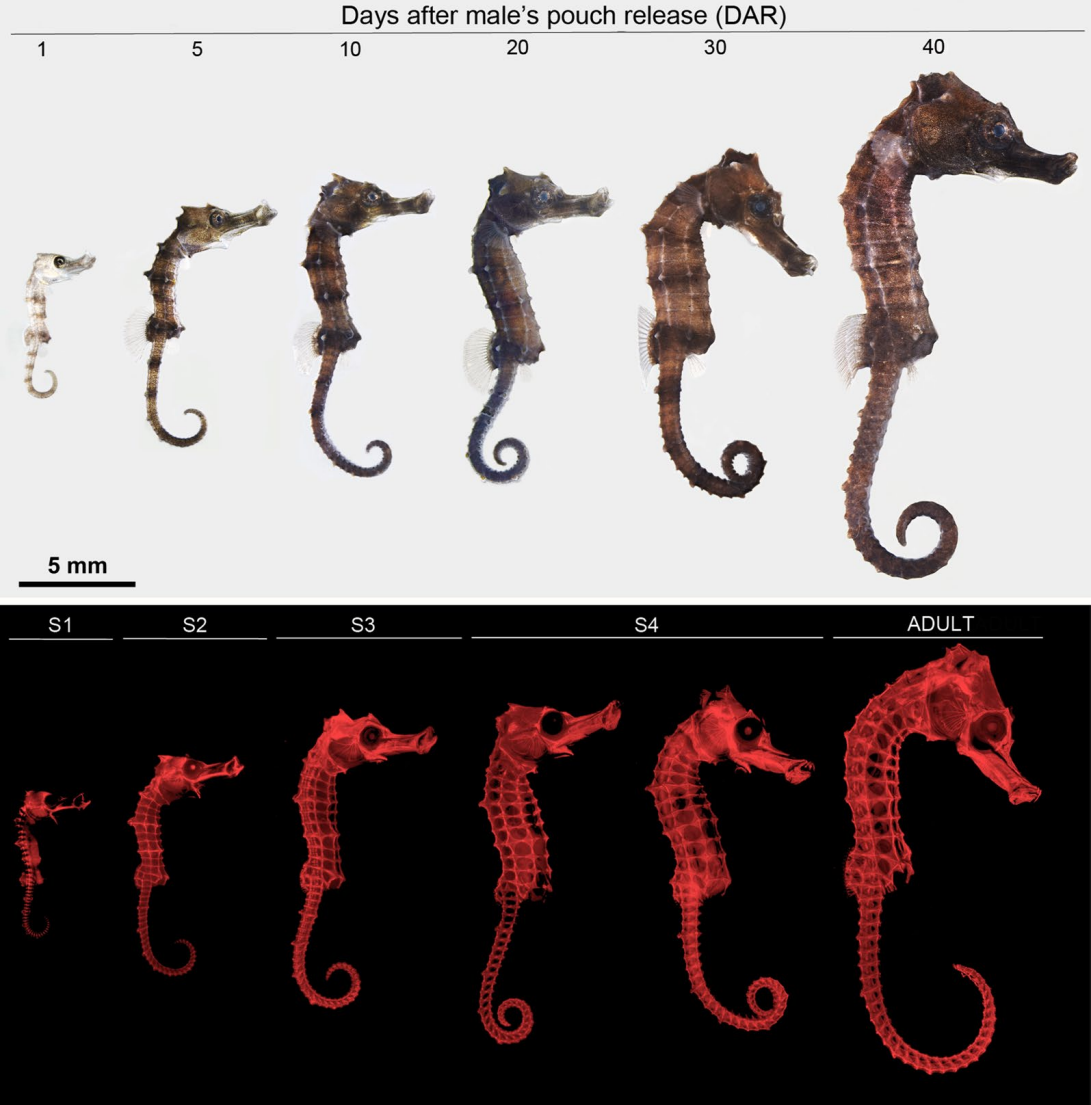
External morphological development of the seahorse *Hippocampus reidi* after male’s pouch release (1, 5, 10, 20, 30 and 40 days after male’s pouch release (DAR)) and developmental stages (S1–S4 and adult stage) under bright-field imaging (up panel) and alizarin red staining (down panel). Seahorse traits including prehensile tail abilities, coronet formation and shift from pelagic to benthic behaviour were observed across development [14]. Scale bar: 5 mm

The external morphological development of *H. reidi* was monitored across development (1, 5, 10, 20, 30 and 40 DAR). Specific developmental stages were stablished according [14] (Fig. 1). Thus, the six ages sampled from 1 to 40 DAR were grouped into five main developmental stages. Newborn seahorses at 1 DAR were included into stage 1 (S1) while 5 and 10 DAR individuals belonged to stage 2 and 3 (S2 and S3), respectively. Stage 4 (S4) comprised seahorses at 20 and 30 DAR. Finally, the more advanced stage, considered as the adult stage, included 40 DAR seahorses [14].

**Fig. 2:**
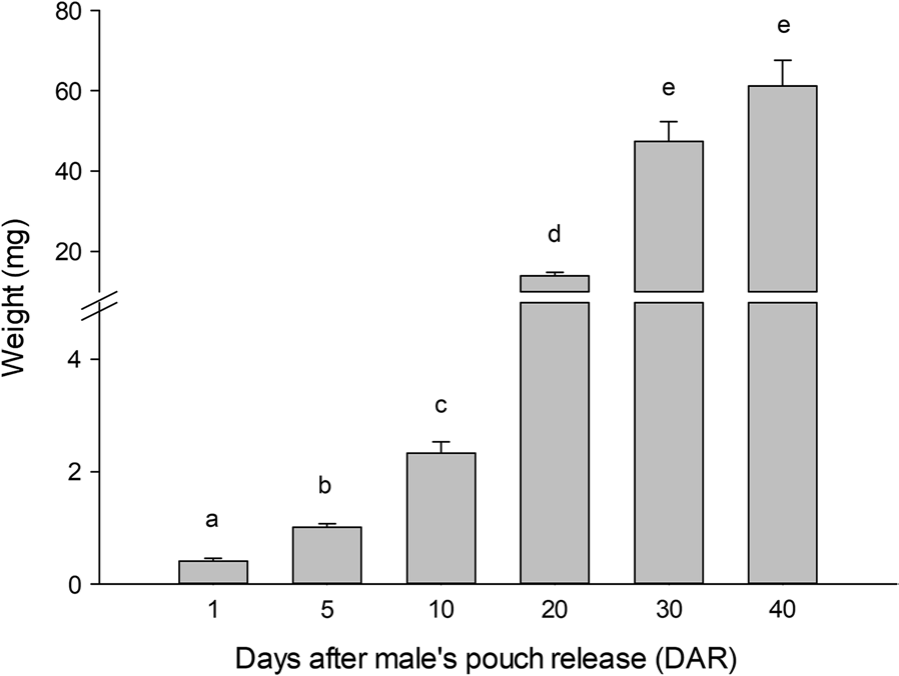
*Hippocampus reidi* growth across development (from 1 to 40 DAR) based on weight measurements. Data are represented as means ± SEM (n = 15). Different lower-case letters denote significant differences (Kruskal–Wallis and pairwise Mann–Whitney U-test (with Bonferroni *p*-value adjustment method) *p* < 0.05)

From birth onwards, seahorses showed a progressive increase in weight (Fig. 2). The highest weight increase was observed at 20 DAR (13.93 ± 0.89 mg) where animals reached almost six times more weight compared to the previous stage at 10 DAR. Later, 30 and 40 DAR seahorses also exhibited a prominent weight increase however, no significant differences were found between these two last age groups (47.37 ± 4.94 mg and 61.19 ± 6.38 mg, respectively).

### Dynamic changes in DNA methylation

The MSAP analysis yielded a total of 233 loci. Of these, 229 were classified as methyl sensitive loci (MSL) and the remaining 4 ones were as non-methyl sensitive loci (NML). All NML were polymorphic loci while the proportion of polymorphic MSL was 95%, and so 217 loci were used for the subsequent analysis. The study of different methylation states (i.e., non-methylated, hemimethylated, internal cytosine methylation and hypermethylated states) across the age groups sampled of *H. reidi* showed a gradual demethylation over the time from 1 to 40 DAR (Table 1). Specifically, between 10 and 20 DAR a change in the internal C methylation dynamic seems to occur. Thus, 20–40 DAR groups displayed a marked loss of internal C methylation status compared with the early age groups (1–10 DAR) (Table 1).

**Table 1:** Frequency (expressed as percentages %) of different DNA methylation states (i.e., unmethylated, hemimethylated, internal cytosine methylation and hypermethylation) at target sequences across different age groups in *H. reidi*

Target state (band pattern)	1 DAR	5 DAR	10 DAR	20 DAR	30 DAR	40 DAR
*Unmethylated (HPA*+*/MSP*+*)*	9.61	18.28	25.33	39.71	41.98	43.30
*Hemimethylated (HPA*+*/MSP-)*	6.0	10.25	12.35	11.70	10.66	10.39
*Internal C methylation (HPA−/MSP*+*)*	35.63	44.10	39.11	17.53	18.02	14.29
*Hypermethylation (HPA−/MSP−)*	47.76	27.37	23.21	31.06	29.34	32.02

Restriction enzyme activity expressed as positive (+) or negative (−)

**Fig. 3:**
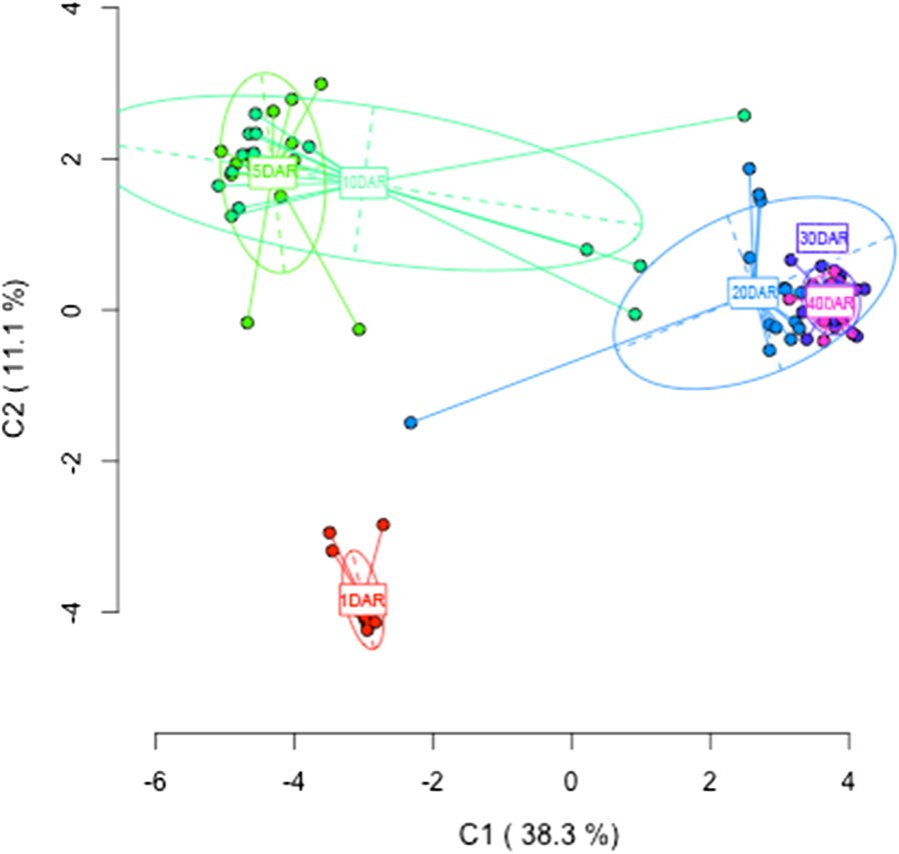
Results from principal coordinate analysis (PCoA) displaying the epigenetic differentiation among six stages of seahorse development as derived from MSAP analysis. The first two coordinates (C1 and C2) are shown with the percentage of global variance explained by them between parentheses. Colored circles represent seahorse individuals from each development stage and the ellipses delimitate the variance of each group (1, 5, 10, 20, 30, 40 DAR). The long axis of the ellipse shows the direction of maximum dispersion and the short axis, the direction of minimum dispersion

**Fig. 4:**
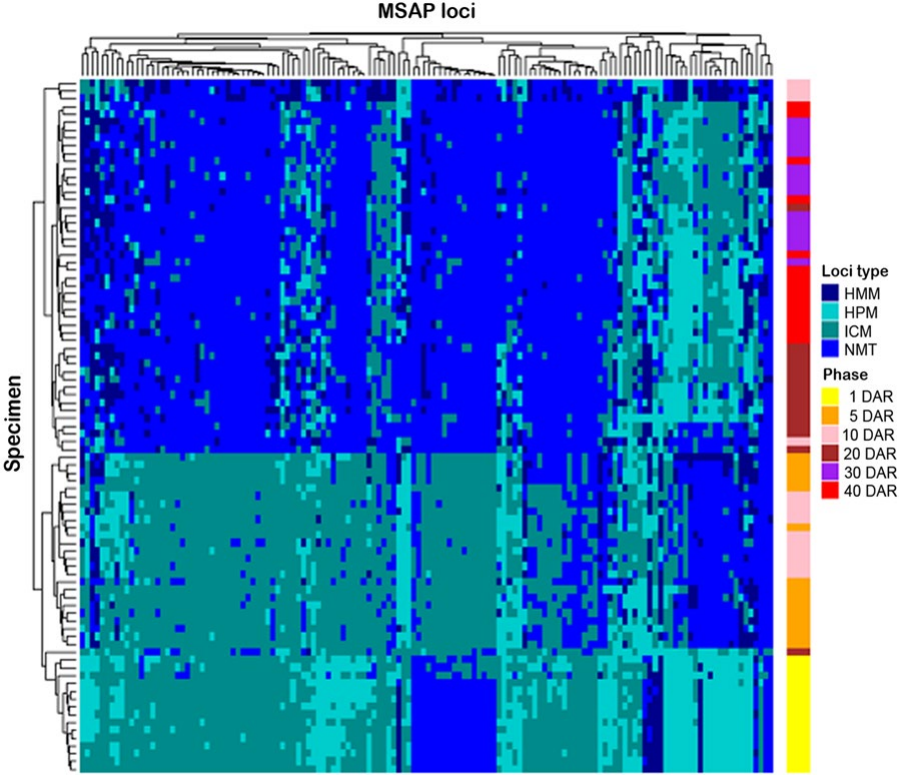
Heatmap of 138 statistically significant methylation-sensitive loci (MSL) among seahorse age groups detected using Fisher’s exact test (*p* < 0.05 after FDR correction). Specimens (rows) and loci (columns) were hierarchically clustered using the unweighted pair group method with arithmetic mean (UPGMA). Loci methylation status (HMM, hemimethylated, HPM, hypermethylated; ICM, internal cytosine methylation; NMT, non-methylated) and the six age groups (1, 5, 10, 20, 30, 40 DAR) are indicated in the right side of the figure. A total of 90 *H. reidi* individuals were analyzed (n = 15 per group)

More in detail, comparisons among age groups by AMOVAs revealed epigenetic variation for MSL (ΦST = 0.5084, p < 0.0001), while no significant genetic differences were found for NML (ΦST = 0.0297, p < 0.0832). As expected, since the seahorses analyzed are siblings from the same progeny, the AMOVA results evidence the genetic homogeneity of the samples, which is also supported by Shannon’s diversity index on the NML (0.1726 ± 0.0461) (mean ± SEM). The DNA methylation pattern differences among groups was further evaluated by PCoA analysis (Fig. 3). The first coordinate (C1) accounts for 38.3% of the global observed variation, allowing to discriminate between three main age groups: one of them corresponding to the 1 DAR group, the second one includes the 5 and 10 DAR groups, and the third one comprises 20, 30 and 40 DAR groups. Moreover, statistical analysis of MSL among age groups by Fisher’s exact test (*p* < 0.05 after applying an FDR correction) identified a total of 138 significant MSAP loci that were represented on a heatmap (Fig. 4). According to PCoA results, these 138 differentially methylated loci were clustered within the heatmap in three major groups exhibiting specific DNA methylation profiles (Fig. 4). From bottom to top, specimens belonging to the 1 DAR age group were clustered together, while specimens from 5 to 10 DAR and 20–30–40 DAR were mostly grouped in two independent clusters. Moreover, we observe that the most MSAP loci are characterized by losing the methylation status across seahorse postnatal development, especially after 10–20 DAR, which supports the results based on the frequencies of the different methylation states in each age group (Table 1). However, this demethylation dynamic over the time is not exactly true for all MSAP loci. For instance, the rightmost cluster on the heatmap including 22 loci showed a different methylation profile. At 1 DAR these loci displayed a high proportion of hypermethylated states, which mainly transitioned to non-methylated loci at 5–10 DAR, and finally they reverted to hypermethylated and internal cytosine methylation states after 20 DAR onwards (Fig. 4).

### dnmt1 and dmnt3b gene expression profiles

**Fig. 5:**
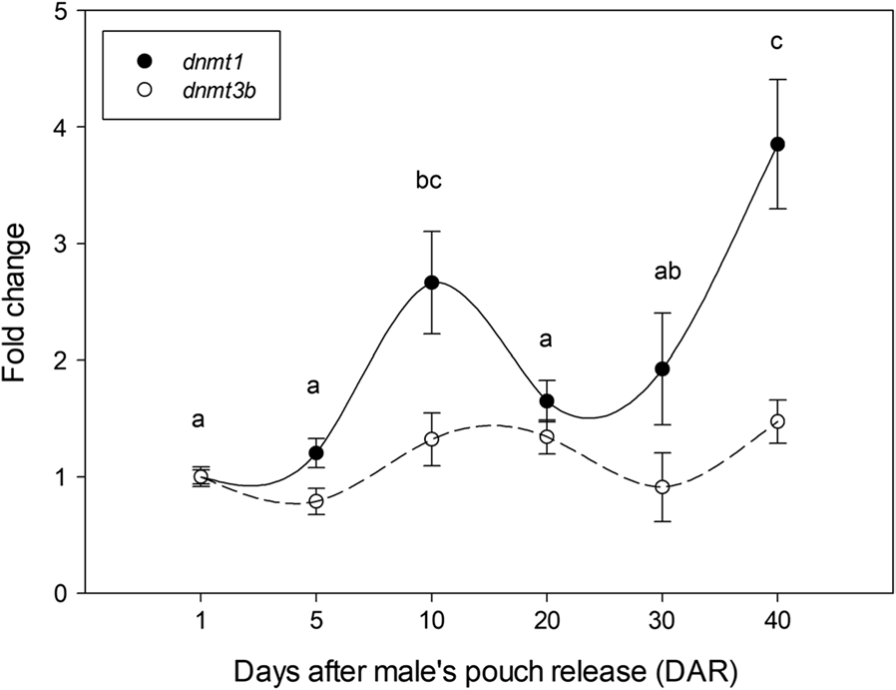
Quantitative real-time PCR (qPCR) analysis of *dnmt1* and *dnmt3b* transcripts through seahorse development after male’s pouch release. The relative expression was determined by qPCR and standardized to *18S*. The results are expressed as mean ± SEM (n = 6) with respect to 1 day after male’s pouch release (DAR), which was set at 1. One-way ANOVA with the Tukey’s post hoc test were performed. Different letters show statistically significant differences (*p* value < 0.05)

Analysis of the *dnmt1* and *dnmt3b* transcript levels were determined for each seahorse age group (1–40 DAR) by using qPCR. A similar gene expression pattern of both DNA methyltransferases *dnmt1* and *dnmt3b* was detected through development (Fig. 5). Specifically, significant statistical differences were found in the *dnmt1* gene expression at 10 and 40 DAR, where *dnmt1* expression level was upregulated. However, no significant differences in the gene expression of *dnmt1* were found at 1, 5, 20 and 30 DAR, exhibiting basal expression levels close to those of *dnmt3b* gene (Fig. 5).

## Discussion

This study has investigated for the first time the involvement of epigenetic mechanisms in the postnatal development in syngnathids, particularly in the seahorse *H. reidi*. Specifically, we found changes in seahorse DNA methylation pattern associated with the early post-natal development and the subsequent transition from planktonic to demersal lifestyle. Global DNA methylation profiles were mainly based on the internal C methylation and hypermethylation at 1 DAR, while seahorses at 5–10 DAR displayed a high proportion of internal C methylation and a transition to non-methylated status started to be noticeable. Later on, from 20 DAR onwards most loci analyzed of the *H. reidi* genome exhibited a clear unmethylated status.

In vertebrates, epigenetic mechanisms participate in the control of the complex gene activation/repression network that promote important developmental processes and life-history events [20, 39]. DNA methylation is one of the key players in the epigenetic control of the genome transcriptional activity [40]. Our results showed a significant demethylation of the *H. reidi* genome between 10 and 20 DAR, when animals start to modify their behavior from pelagic to demersal lifestyle [7, 8] and certain morphological and physiological features are supposed to be acquired [13, 14]. In this context, previous studies have also evidenced the important effect that genome-wide DNA methylation changes have in other marine organisms with complex life-cycles involving developmental and ecological transitions. In European eel, transitions between developmental stages and new habitats (i.e., freshwater or seawater environments) were associated with different DNA methylation patterns analyzed by MSAP in gills, which is a crucial tissue for osmoregulation [28]. Similarly, smoltifying salmonids such as rainbow trout [25] and brown trout [24] showed DNA methylation-mediated epigenetic differences in branchial tissue. Other studies in sea lamprey [29] and Japanese flounder [30] specifically reported an epigenetic regulation of genes involved in metamorphosis including those related to morphogenesis or water balance, which are necessary for juvenile adaptation to new environments. Although higher methylation rates are usually expected as vertebrate development progresses [29, 41], our data come to light a mostly methylated *H. reidi* genome during early postnatal larval stages (1–5–10 DAR) while an unmethylated genome would be linked to juvenile (20–30 DAR) and adult stages. Those results are further supported by *dnmt1* gene expression analysis and so, after 10 DAR a down-regulation of methyltransferases transcripts agreed with the demethylation dynamic observed for the investigated loci. Nevertheless, a different DNA methylation dynamic was also detected for a low proportion of MSAP loci across seahorse development. Here, 10–20–30 DAR age groups presented an increase of the methylation rates that could be associated to the up-regulation of methyltransferases expression during late development.

The S3 developmental stage in *H. reidi* has previously been defined from 9 to 18 DAR and identified as the one in which great biological shifts take place including anatomical changes (e.g., new intestine disposition and loops formation; development of prehensile tail abilities, among them) as well as changes in the planktonic-to-benthonic behavior [14]. According to our data, seahorses show a progressive weight increase from birth and during the planktonic phase that approximately persists until 18–20 DAR [14], when seahorses seem to be ready for settlement. Around the 20 DAR, seahorses improved their prehensile tail abilities and started to change their behavior as they spent more time near the bottom holding onto a variety of artificial substrates. Therefore, we hypothesized that DNA methylation and the subsequent variations in the global gene expression patterns might be part of the adaptation mechanisms associated to the postnatal development and ecological transition to the demersal habitat in *H. reidi*. Nevertheless, if the DNA methylation changes could be a consequence rather than a cause of the seahorse postnatal development is unknown. In this context, some studies have argued that variation in the DNA methylome dynamic could be a secondary event during aging [42] and disease [43], not being the cause that triggers those physiological or pathological processes.

The extraordinary life cycle of seahorses along with their unique biological and ecological traits deserve to be understood in detail. Moreover, the seahorse *H. reidi* is one of the most demanded species for ornamental aquarium trade and traditional Chinese medicine and, therefore, managing the seahorse overexploitation in the wild is crucial [32, 33]. Syngnathid aquaculture is a promising strategy for animal conservation, and investigations focused on the molecular factors that lead optimal growth and survival will contribute to a successful farming [18]. Our global DNA methylation studies across *H. reidi* postnatal development provide new knowledge to achieve this goal as epigenetic mechanisms seem to be necessary for seahorse settlement in the bottom of the sea. Nevertheless, if these epigenetics mechanisms come from internal or they are initiated via external environmental cues should be further investigated [44].

## Conclusions

Our study provides new insight into the potential functional mechanisms involved in postnatal development and the transition from planktonic to benthonic lifestyle observed in seahorses. Our results demonstrate that DNA methylation dynamics of the loci analyzed via a methylation-sensitive amplified polymorphism (MSAP) technique could be associated with postnatal development and the ecological transition to a new habitat in seahorses. We have also shown that DNMTs gene expression levels are regulated and agreed with the observed DNA methylation dynamics. This is a preliminary study reporting the global DNA methylation patterns during developmental and ecological transitions of a captivating group of fishes with unique biological and ecological traits in the animal kingdom. However, it remains to be seen whether these epigenetic mechanisms come from either internal or external environmental cues or probably a combination of both.

## Material and methods

### Seahorse culture and sampling

*Hippocampus reidi* broodstock were reared in 500 L aquaria tanks provided with plastic seagrass and ropes for attachment in a semi-opened seawater system. Culture conditions mimicked natural regimes from tropical areas. Temperature ranged 26–27 °C and photoperiod was 10L:14D or 14L:10D for winter or summer, respectively. Seahorses breeders were fed three times daily on cultivated adult *Artemia* and frozen mysids.

Newborn seahorses used for this study were obtained from a single batch. Larvae were maintained in 30 L tanks (in duplicate) for 40 days at about 5 juveniles litre^−1^. The seahorses were fed on cultivated copepods *Acartia tonsa* for the first week after pouch release, on a mixture of copepods and *Artemia* nauplii until day 10 and on *Artemia* (nauplii and metanauplii) [45].Seahorses were sampled at 1, 5, 10, 20, 30 and 40 days after male’s pouch release (DAR) and the specific developmental stage was determined [14]. Briefly, for each age group, 24 individuals were randomly collected, euthanized using tricaine methanesulfonate (MS-222) and weighed (wet weight). Further information on the experimental system and rearing is available in [46, 47].

### DNA and RNA isolation

For DNA extraction, 15 individuals at 1, 5, 10, 20, 30 and 40 DAR were conserved in absolute ethanol at 4 °C. Samples were sliced and homogenized. Genomic DNA was extracted from resulting homogenized material using the NZY Tissue gDNA Isolation Kit (Nzytech) according to the manufacturer’s indications. DNA quality was verified by electrophoresis on 1% agarose gels. Subsequently, DNA concentration was quantified on a Nanodrop 1000 spectrophotometer (Thermo Fisher Scientific) and samples were adjusted to 25 ng/μl.

For RNA isolation, 6 individuals per age group were fixed in RNA later for 24 h at 4 °C and conserved at − 80 °C. Total RNA was extracted and purified using the RNeasy Midi Kit (Qiagen) for the samples with a tissue amount greater than 30 mg and RNeasy Mini Kit (Qiagen) for those less than 30 mg. RNA concentration was quantified on a Nanodrop 1000 spectrophotometer (Thermo Fisher Scientific). Subsequently, cDNA was synthesized according to the Maxima First Strand cDNA Synthesis (Thermo Fisher Scientific) protocol with 100 ng of RNA.

### External morphological development

Seahorses (n = 3) at 1, 5, 10, 20, 30 and 40 DAR were quickly fixed in 4% paraformaldehyde (PFA; Alfa Aesar, ThermoFisher, Germany) in 1X PBS overnight at 4 °C. Fixative was removed washing three times with PBS for 15 min. Seahorses were then imaged under bright-field light. Pigmentation was removed with H_2_O_2_ (6% final concentration), treatment time was empirically determined based on the age (from 2 h or less for early stages until overnight treatment for later ones). After washing H_2_O_2_ 3 times with fresh water, seahorses were dehydrated in 50% ethanol for 1 h and stained for 1 h at room temperature with 0.01% alizarin red S (AR-S) prepared in 70% ethanol (from a stock solution 0.5% AR-S in H_2_O and adjusting pH to 7.4 with Tris–HCl). AR-S was washed twice with 70% ethanol for 5 min. Seahorses were then imaged under fluorescent light using Texas Red filter. All images were captured using a Leica M165FC stereomicroscope (Leica Microsystems, Germany) equipped for epifluorescence together with a DFC310Fx camera and LAS software. Images were processed with Adobe Photoshop.

### Methylation-sensitive amplified polymorphism (MSAP)

Genome-wide DNA methylation analysis was carried out by following the MSAP protocol described by [48], which is a modified version from [49] and [50] methodologies. The MSAP technique is based on the use of two cytosine methylation-sensitive restriction endonucleases, HpaII and MspI, to detect DNA methylation fingerprints. HpaII and MspI are isoschizomers which recognize the same sequence (5'-CCGG sites) but display differential sensitivity to DNA methylation. HpaII is sensitive to internal cytosine methylation, whereas MspI is sensitive to external cytosine methylated in hemimethylated targets (i.e., sites methylated only at one DNA strand) (http://rebase.neb.com) [51]. Also, HpaII and MspI are sensitive to hypermethylated sites (*i.e.,* both at the internal and external cytosines), whereas unmethylated targets are digested by both enzymes. Therefore, the restriction profiles for each single enzyme allow the assessment of the methylation state of the target fragments.

For each sample, 50 ng of DNA were independently digested with both EcoRI/MspI and EcoRI/HpaII enzyme combinations (New England Biolabs) and ligated to specific adaptors in parallel reactions as described in [24]. Afterwards, restriction fragments were amplified through two consecutive PCR reactions: pre-selective and selective PCR amplifications as detailed in [24]. Primer sequences used for pre-selective and selective PCR reactions are available in [48]. Finally, 2 μl of the labelled MSAP PCR products was combined with 15 μl of HiDi formamide and 0.5 μl of GeneScan 500 ROX size standard (Applied Biosystems). Samples were heat-denatured at 95 °C for 5 min and cooled on ice for 5 min. Samples were fractionated on an ABI Prism 3100 Genetic Analyzer (Applied Biosystems) at 15 kV for 6 s and at 15 kV for 33 min at 66 °C. MSAP restriction profiles were scored using GeneMapper v.3.7 software (Applied Biosystems). DNA fragments less than 100 bp in length, longer than 500 bp or less than 70 RFU (Relative Fluorescent Units) were excluded from the analysis due to low levels of reproducibility.

### Quantitative real-time PCR (qPCR)

qPCR analysis was performed using the Maxima SYBR Green/ROX qPCR Master Mix (2X) (Thermo Fisher Scientific). cDNA samples (n = 36) were amplified by triplicate containing 12.5 μl of Maxima SYBR Green/ROX qPCR Master Mix (2X) solution, 0.5 μl 0.2 μM of each primer, 10.5 μl nuclease free water and 1 μl of cDNA template. qPCR reactions were analyzed on a 7500 Fast Real-Time PCR System (Applied Biosystems) with the following cycling conditions: initial denaturation at 95 °C for 10 min followed by 40 cycles at 95 °C for 15 s and 60 °C for 1 min. Expression levels of *dnmt1* and *dnmt3b* genes were assessed. Relative mRNA expression levels normalized to the housekeeping *18S* ribosomal gene were analyzed using the efficiency calibrated method as previously described [52]. Primer sets used for each gene are listed in Table 2.

**Table 2:** Primer sequences used for quantitative real time PCR gene expression analysis in *H reidi*

Genes	Forward primer sequence (5′-3′)	Reverse primer sequence (5′-3′)
*dnmt1*	CACCACTGTCACCAATCCTG	GGTACGTATCGGGGAAACCT
*dnmt3b*	TTTGCGTGCTGAAAGATGTC	ACTCCATGGCACTGTTGTTG
*18S*	CGCGAGATTGAGCAATAACA	TACTGGGAATTCCTCGTTGG

### Data analyses and statistics

MSAP restriction profiles were assessed following a band-based strategy [53] for products from EcoRI-HpaII and EcoRI-MspI primers, independently. The resulting presence/absence matrices were integrated and analyzed with the R package MSAP [54]. Then, every band was scored as follows: present in both EcoRI-HpaII and EcoRI-MspI products (1/1), denoting non-methylated state (NMT); present only in either EcoRI-HpaII (1/0) or EcoRI-MspI (0/1) products, corresponding to hemimethylated (HMM) or internal cytosine methylation state (ICM), respectively. The absence the both bands (0/0) was considered as hypermethylated state (HPM). Loci were classified as methylation-sensitive loci (MSL) versus the non-methyl sensitive loci whether the observed proportion of methylated states was greater than 5% (error rate-based threshold). Only MSL showing at least two occurrences of each state (i.e., polymorphic loci) were selected for further analysis.

The study of the DNA methylation pattern differences among age groups was performed by analyses of molecular variance (AMOVA) and principal coordinates analysis (PCoA). Genetic variation was estimated using Shannon’s diversity index. Furthermore, locus-specific methylation differences between groups were assessed using Fisher’s exact test. After *p*-value adjustment by false discovery rate (FDR) correction method [55], only MSL with a *p* < 0.05 were selected. Estimates of relationships among selected loci from the four categories (NMT, HMM, ICM or HPM) were computed by Gower’s Coefficient of Similarity. The resulting distance matrix was used to perform hierarchical clustering using unweighted pair group method with arithmetic mean (UPGMA) method and visualized as a heatmap matrix with the R “ComplexHeatmap” package [56].

For growth analysis based on weight measurements and quantitative gene expression analyses, data were expressed as mean ± standard error of the mean (SEM). Non parametric Kruskal–Wallis followed by pairwise Mann–Whitney U-test (with Bonferroni *p*-value adjustment method) was performed for comparison between postnatal weight data. Multiple comparisons between quantitative gene expression data were evaluated by one-way ANOVA with the Tukey’s post hoc test. Data are statistically similar if they shared at least one letter. A *p *value < 0.05 (letters) was considered statistically significant. Statistical analyses and figures were performed in R [57] with R Studio (R statistical software v3.3.2) and SigmaPlot 12.0.

## Data Availability

The datasets used and analysed during the current study are available from the corresponding authors on request.
